# Identification of key pathways and mRNAs in interstitial cystitis/bladder pain syndrome treatment with quercetin through bioinformatics analysis of mRNA-sequence data

**DOI:** 10.18632/aging.205682

**Published:** 2024-03-22

**Authors:** Guang Wang, Pei Li, Si-Wei Su, Rui Xu, Zi-Ye Huang, Tong-Xin Yang, Jiong-Ming Li

**Affiliations:** 1 Department of Urology, The Second Affiliated Hospital of Kunming Medical University, Kunming, Yunnan 650101, P.R. China

**Keywords:** interstitial cystitis/bladder pain syndrome, quercetin, mRNA sequence, high performance liquid chromatography, Lpl

## Abstract

Background: Interstitial cystitis/bladder pain syndrome (IC/BPS) is a chronic condition with painful bladder. At present, the pathogenesis of IC/BPS is still unknown. Quercetin (QCT) is a kind of natural flavonoid with wide sources and multiple biological activities. The purpose of this study was to explore the effects of QCT on mRNA expression and related regulatory signal pathways in IC model rats.

Methods: LL-37 was used to induce the IC/BPS model rats. 20 mg/kg QCT was injected intraperitoneally into IC/BPS rats. ELISA, HE, Masson and TB staining were used to evaluate the level of inflammation and pathology. The concentration of QCT in rats was detected by HPLC. The mRNA sequencing was used to detect the differentially expressed (DE) mRNA in each group. The over-expression experiment of Lpl was carried out in IC/BPS model rats.

Results: QCT treatment significantly decreased the level of MPO, IL-1β, IL-6 and TNF-α induced by LL-37 in rats, and alleviated bladder injury and mast cell degranulation. There were significant differences in mRNA sequencing data between groups, and the hub gene Lpl were screened by Cytohubba. The expression of Lpl was downregulated in IC/BPS rats. QCT intervention promoted Lpl expression. Overexpression of Lpl reduced the bladder injury induced by LL-37, increased GAG level and decreased the expression of MPO, IL-1β, IL-6 and TNF-α.

Conclusion: In this study, we provided the DE mRNA in IC/BPS rats treated with QCT, the signaling pathways for DE enrichment, screened out the hub genes, and revealed that Lpl overexpression alleviated IC/BPS model rats.

## INTRODUCTION

Interstitial cystitis/bladder pain syndrome (IC/BPS) is a chronic inflammatory disease, with clinical manifestations of urgency, nocturia and pelvic pain, which seriously affecting the health and life quality of patients. The incidence of IC/BPS in women is significantly higher than that in men, and the incidence ratio of is about 1:9 [1]. At present, it is found that the pathogenesis of IC/BPS is related to occult infection, mast cell activation, neuritis and glycosaminoglycan [2], but the specific mechanism remains to be studied. Therefore, it is of great significance to explore the specific mechanism of IC/BPS for its clinical treatment.

Quercetin (QCT), whose chemical name is 3,5,7,3′,4′-pentahydroxy flavone, is a natural flavonoid compound. Quercetin has two benzene rings (ring A and ring B), which are connected by a 3-carbon chain to form a closed pyran ring (ring C) with five hydroxyl groups. Its glycosylation can occur on any hydroxyl group, and various quercetin glycoside forms can be produced by combining with glucose, xylose or rutin sugar [3]. Quercetin has chemical preventive and therapeutic effects on many diseases, and its activities are shown as antioxidant, anticancer, antihypertensive, antidiabetic, anti-inflammatory, antiviral, anti-Alzheimer’s disease, cardiovascular protection, neuroprotection, anti-aging and improving immunity [4–6].

Regulating inflammation is one of the most significant functions of quercetin. It has been proved in many studies that quercetin can inhibit inflammatory cytokines and inflammatory enzymes *in vivo* and *in vitro*, and it can act on different cell types in animal and human models. Studies have confirmed that 50 μm quercetin can significantly inhibit the production of IL-6, MCP-1, IP-10, RANTES, GM-CSF, G-CSF, TNF-α, LIF, LIX and VEGF, and can also inhibit the release of calcium from mouse macrophages induced by dsRNA. It is concluded that quercetin can inhibit the release of inflammatory factors in macrophages induced by dsRNA through calcium homeostasis pathway [7]. Some studies have found that quercetin has a protective effect on IC/BPS induced by stress, and quercetin can improve the morphological integrity of urothelium, the increase of inflammatory cells and the proliferation of mast cells [8]. Therefore, quercetin has certain therapeutic potential in the therapeutic prospect of interstitial cystitis.

In this study, we used cathelicidin LL-37 to induce IC/BPS model rats, and intraperitoneal injection of QCT as an intervention factor. Then, the concentration of QCT in rat plasma and urine was detected by high performance liquid chromatography (HPLC). Enzyme-linked immunosorbent assay (ELISA), hematoxylin-eosin (HE), Masson and toluidine blue (TB) staining were used to observe the effect of QCT on IC/BPS rats. Meanwhile, mRNA sequencing in rat bladder tissue was used to further explore the molecular mechanism of QCT regulating IC/BPS.

## MATERIALS AND METHODS

### Experimental rats and ethical approval

40 specific pathogen free female Sprague-Dawley (SD) rats (200 ± 20 g, *n* = 5) were provided by Hunan SiLaiKeJingDa Experimental Animal Co., Ltd. (SCXK (Xiang) 2019-0004). Rats were raised in the environment with 22 ± 1°C, 50 ± 5% humidity and 12-h light/dark cycle, and the rats had free access to water and food. Our study was approved by the Laboratory Animal Ethics Committee of the Kunming Medical University (IACUC Issue No: kmmu2021153). All experiments were performed in accordance with relevant guidelines and regulations. This study is reported in accordance with ARRIVE guidelines.

### Construction of IC/BPS model

After feeding for one week, SD rats were randomly divided into 5 groups (Ctrl group *n* = 10, IC/BPS group *n* = 10, IC/BPS+QCT group *n* = 10, IC/BPS+QCT+pcDNA-NC group *n* = 5, IC/BPS+QCT+pcDNA-Lpl group *n* = 5). 150 μL LL-37 was slowly injected into rat bladder through catheter on the first, fourth and seventh day to establish rat model. On the eighth day, HE and Masson staining were used to identify the successful establishment of IC/BPS model.

### QCT treatment and plasmid injection

One day after the construction of IC/BPS model, 20 mg/kg QCT was intraperitoneally injected to rats [9]. Then 20 mg/kg QCT was injected on the third and sixth day. Rats in the Ctrl groups were given the same amount of normal saline.

Then two hours later, the rats in IC/BPS+QCT+pcDNA-NC group and IC/BPS+QCT+pcDNA-Lpl group was injected pcDNA-NC and pcDNA-Lpl plasmid through tail vein, and then injected with plasmid again on the third and sixth days after the model was established.

### Sample collection

Before the rats were sacrificed, the peripheral blood was collected, and serum was collected from whole blood by centrifugation for ELISA assay. Another part of whole blood from the IC/BPS+QCT group was used for HPLC assay. The 24-h urine volume (The seventh day) and the residual urine after bladder removal in the IC/BPS+QCT group were collected and used for HPLC assay.

The bladder tissues were divided longitudinally into 3 parts. One portion of the tissue was fixed with 4% paraformaldehyde for morphological testing. One portion of the tissue was frozen in liquid nitrogen for mRNA sequencing. One portion of the tissue was fixed with 2.5% glutaraldehyde and used for transmission electron microscopy.

### HPLC assay

After acidification of 100 μL plasma or urine with 20% formic acid solution, 10 μL ascorbic acid was added to prevent oxidation of QCT. A total of 300 μL acetonitrile was added into the sample to extract QCT. After the QCT was centrifuged at 12000 r/min for 10 min, the supernatant was taken, and methanol was added into the residue to repeat the above extraction steps again. The mixed solution was air-dried by nitrogen flow, reconstituted by adding methanol solution, and filtered through a 0.22 μm microporous membrane. The samples were analyzed by Shimadzu LCMS-8040 triple and four-stage liquid chromatography-mass (LC-MS) spectrometry (Shimadzu, China).

### ELISA

ELISA kit detected the concentration of IL-1β, IL-6, MPO and TNF-α (Shanghai Enzyme-linked Biotechnology Co., Ltd., China). According to the instruction of ELISA kit, the standard (50 μL) and sample (40 μL Sample diluent and 10 μL sample) were added to the standard holes and sample holes of microtiter plate, respectively. The culture plates were incubated at 37°C in 5% CO_2_ for 30 min and then rinsed with washing solution. Then 50 μL enzyme-labeled reagent was added into each well, and the samples were incubated for 30 min and washed. Then 50 μL color developer A and color developer B were added, mixed, and stained in the dark for 10 min. After the reaction was stopped by adding stop solution, the absorbance density (OD) was measured at 450 nm by enzyme-labeled instrument (Molecular, CA, USA).

### HE

The bladder tissue fixed with paraformaldehyde was placed in an embedding box, and xylene (XiLong Chemical Co., Ltd., China) was added for transparency after dehydration with ethanol (XiLong Chemical Co., Ltd., China). The tissue samples were cut into 5 μm thick sections after paraffin embedding. The paraffin sections were rinsed with dewaxing, hydration and distilled water, stained with hematoxylin (Sigma, MO, USA) for 10 min, and differentiated 2 s using 1% hydrochloric acid ethanol after rinsing. Subsequently, it was stained with eosin (Sigma, MO, USA) for 5 min. After stopping coloration, it was dehydrated with ethanol and transparent by adding xylene. The slices were sealed with neutral glue (Shanghai Aladdin, China). The staining results were observed by a microscope (JiNan DanJiEr Electronics Co., Ltd., China).

### Masson

After dehydration, wax dipping and embedding of the fixed bladder tissue, the sections were placed in a 65°C incubator for 30 min. After dewaxing and hydration, the samples were stained with Weigert iron hematoxylin (Beijing Solarbio Science and Technology Co., Ltd., China) for 10 min. After differentiation with acidic ethanol (Xilong Chemical Co., Ltd., China), the sections were rinsed and stained with Masson Blue Solution (Beijing Solarbio Science and Technology Co., Ltd., China) for 3 min. After washing, Ponceau S was added for staining for 10 min. After the sections were rinsed again, they were made transparent by xylene, sealed with neutral glue and then observed under a microscope.

### TB staining

Tissue samples were fixed, paraffin embedded, sectioned and then dewaxed and hydrated with gradient ethanol. After the slices were washed with distilled water, TB staining solution (Beijing Solarbio Science and Technology Co., Ltd., China) was added for staining for 5 min. The slices were rinsed, added with 0.5% acetic acid to differentiate into blue positive cells, dehydrated with gradient ethanol, and made transparent with xylene for 30 min. After the slices were sealed with neutral glue, then the scanning electron microscope was used to observe the results.

### Electron microscopy

The bladder tissue was first fixed with glutaraldehyde solution and then treated with the addition of 1% OsO_4_. After gradient ethanol dehydration, Quetol 812 resin was added to fix the bladder tissue again. After sectioning, the sections were stained with saturated uranyl acetate in 50% ethanol solution for 30 min and 0.1% lead citrate solution for 15 min at room temperature, washed and observed under a transmission electron microscope.

### RNA extraction

The bladder tissue of rats was taken, cut into pieces with scissors and ground with liquid nitrogen. TRIzol reagent was added and mixed for complete lysis, and the supernatant was taken after centrifugation. After chloroform was added and shaken for 30 s, isopropanol and ethanol were added, respectively. The supernatant was discarded after centrifugation to obtain RNA. The OD value was detected by microplate reader.

### Library construction

The transcription build library kit was NEBNext^®^ Ultra™ RNA Library Prep Kit for Illumina^®^. The first strand of cDNA was synthesized in M-MuLV reverse transcriptase system with segmented mRNA as template and random oligonucleotide as primer. Then RNA strand was degraded by RNaseH, and the second strand of cDNA was synthesized from dNTPs in DNA polymerase I system. The purified double-stranded cDNA was repaired at the end, poly (A) tail was added, and the sequencing adapter was connected. AMPure XP Beads were used to screen 250–300 bp cDNA. PCR amplification was performed, and AMPure XP beads were used to purify PCR products. Finally, the library was obtained.

### mRNA-sequence

The Qubit 2.0 was used for initial quantification, and the library was diluted to 1.5 ng/μL. Agilent 2100 was used to test the distribution of inserts in the library. After the inserts met the expectation, RT-qPCR was performed to accurately quantitate the effective concentration of the library (the effective concentration of the library is higher than 3 nM) to ensure the quality of the library. For qualified libraries, a minimum of 6G of data was measured for each sample using the Illumina NovaSeq PE150.

### Raw data filtering

To reduce data noise, unqualified sequential reads filters were performed on the original FastQ data, including filtered reads with dropped adapter, reads containing N (N indicates that no base information could be determined) and low-quality reads (Qphred ≤20 reads with bases accounting for more than 50% of the entire read length). The raw data were subjected to the above-mentioned filtering processing and becomes clean data for bioinformatics analysis [10].

### Functional and pathway enrichment and construction of protein–protein interaction (PPI) network

The differential gene sets were enriched in the Gene Ontology (GO), Kyoto Encyclopedia of Genes and Genomes (KEGG) and Reactome databases by the R package “cluster profiler” [11]. Protein-protein interaction (PPI) network was constructed by STRING and further visualized by Cytoscape.

### Data availability

The mRNA sequencing raw data generated and analysed during the current study are available in the NCBI SRA repository (Sequence Read Archive; https://www.ncbi.nlm.nih.gov/sra/PRJNA898519). The communication author can be contacted for more information on the nine mRNA sequencing data presented herein.

### Immunohistochemistry (IHC)

Rat bladder tissue was fixed with 4% paraformaldehyde, embedded in paraffin and sliced. The slices were baked in an oven at 60°C for 30 min, and then dewaxed. Citric acid buffer (pH = 6.0) repaired antigen. The histochemical pen drew a circle on the section to prevent the liquid from leaking out during the dyeing process, and the section was sealed with 5% goat serum for 1 h. The slices were incubated with primary antibody (Lpl, 1:100) and secondary antibody (Lpl, 1:200) respectively, and reacted with DAB chromogenic solution for 1 min. The nuclei were stained with hematoxylin. The slices were treated alcohol and xylene, then sealed with neutral glue, and observed under a microscope.

### Western blot

The total protein was extracted RIPA lysate (Beyotime), and it was denatured by boiling at 100°C for 10 min. IMPLEN ultra-micro spectrophotometer was used to detect the protein concentration. 80 μg samples were subjected to SDS-PAGE gel electrophoresis, and then transferred to PVDF membrane. PVDF membrane (Millipore, MA, USA) was sealed with 5% bovine serum albumin (Solarbio) for 2 h, rinsed with TBST, and then incubated with primary antibody and secondary antibody respectively. Enhanced chemiluminescence (Applygen, China) was used to show protein bands.

### Statistical analysis

All data were analyzed by R software (version 4.2.2) or GraphPad Prism 8.0. One-way ANOVA was used to analyze the difference among multiple groups. The DESeq2 package (version 1.38.3) of R software is used for Wald test of sequencing data [12]. The *p*-values are calculated based on Fisher test of negative binomial distribution. The adjusted *p*-values and FDR values are corrected by the BH algorithm for multiple hypothesis testing. *P* < 0.05 was considered statistically significant. In the statistical analysis of mRNA sequencing results, when *P*.adj <0.05 it is considered to be statistically significant.

## RESULTS

### The concentration of QCT in the urine and plasma of rats

**Figure 1:**
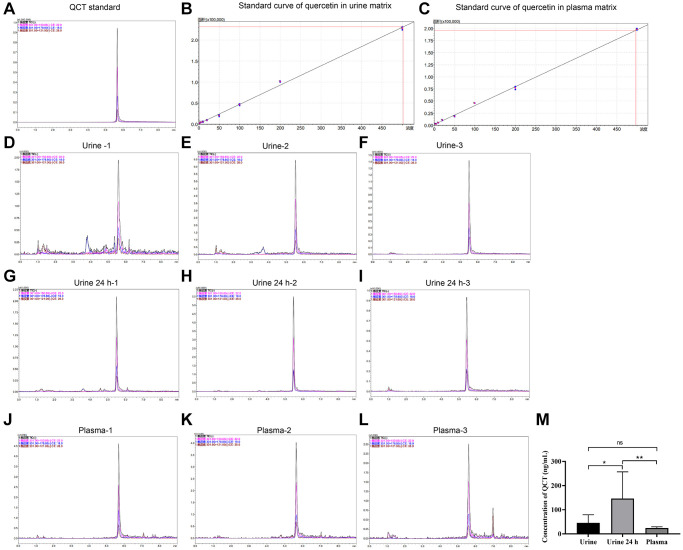
**HPLC detected the concentration of QCT.** (**A**) The HPLC chromatogram of QCT standard. (**B**) The standard curve of QCT in urine matrix. (**C**) The standard curve of QCT in plasma matrix. (**D**) The HPLC chromatogram of QCT in Urine-1 group. (**E**) The HPLC chromatogram of QCT in Urine-2 group. (**F**) The HPLC chromatogram of QCT in Urine-3 group. (**G**) The HPLC chromatogram of QCT in Urine 24 h-1 group. (**H**) The HPLC chromatogram of QCT in Urine 24 h-2 group. (**I**) The HPLC chromatogram of QCT in 24 h-3 group. (**J**) The HPLC chromatogram of QCT in plasma-1 group. (**K**) The HPLC chromatogram of QCT in plasma-2 group. (**L**) The HPLC chromatogram of QCT in plasma-3 group. (**M**) QCT concentration statistics of each group. ^*^P < 0.05, ^**^P < 0.01. Abbreviation: ns: no significant difference.

To investigate the mRNA expression in rats after QCT treatment, we first analyzed the concentration of QCT in plasma and urine of rats after QCT treatment by HPLC. In the QCT standard, (M-H)^−^ was used as the precursor ion, and the characteristic fragment ions were 150.85, 178.80, and 121.00, among which the quantitative ion was 150.85, and the retention time was 5.577 min, as shown in Figure 1A. Then, the standard curve of QCT in the plasma and urine is constructed respectively (Figure 1B, 1C and Table 1). It was confirmed that the retention time of QCT in plasma and urine was the same as that of QCT standard (Figure 1D–1L and Table 2). The concentration of QCT in 24-hour urine group was higher than that in the urine group and plasma group (Figure 1M), but the level of QCT in the urine group was not significantly different from that in the plasma group. These results showed that after injection of QCT, most QCT was excreted through the urinary system, and a small part was absorbed by the digestive system and entered the blood.

**Table 1:** QCT standard curve and the related parameters.

**Matrix**	**Linear equation**	**R**	**LOQ (ng/mL)**	**LOD (ng/mL)**	**Linearity range (ng/mL)**
Plasma	Y = 391.585X + 1536.87	0.999	2	1	2–500
Urine	Y = 458.472X + 1113.26	0.998	2	1	2–500

Abbreviations: QCT: Quercetin; LOQ: limit of quantification; LOD: limit of detection.

**Table 2:** The concentration of QCT in urine and plasma of rats.

**Sample**	**Retention time (min)**	**Concentration (ng/mL)**
QCT Standard	5.577	
Urine-1	5.680 ± 0.0148	11.8093 ± 1.9460
Urine-2	5.5240 ± 0.0123	37.2007 ± 1.9229
Urine-3	5.4947 ± 0.0032	87.8363 ± 3.3362
Urine 24 h-1	5.4853 ± 0.0155	115.7800 ± 7.4888
Urine 24 h-2	5.4580 ± 0.0087	285.5770 ± 9.9292
Urine 24 h-3	5.4343 ± 0.0105	37.7730 ± 20.6362
Plasma-1	5.6947 ± 0.0163	28.0397 ± 1.6488
Plasma-2	5.6297 ± 0.0119	26.6587 ± 2.2161
Plasma-3	5.5990 ± 0.0060	17.3923 ± 2.1748

Urine: The group of residual volume after bladder removal. Urine 24 h: The group of urine volume collected within 24 h. Plasma: The group of plasma of rats.

### The effect of QCT on IC/BPS rats

**Figure 2:**
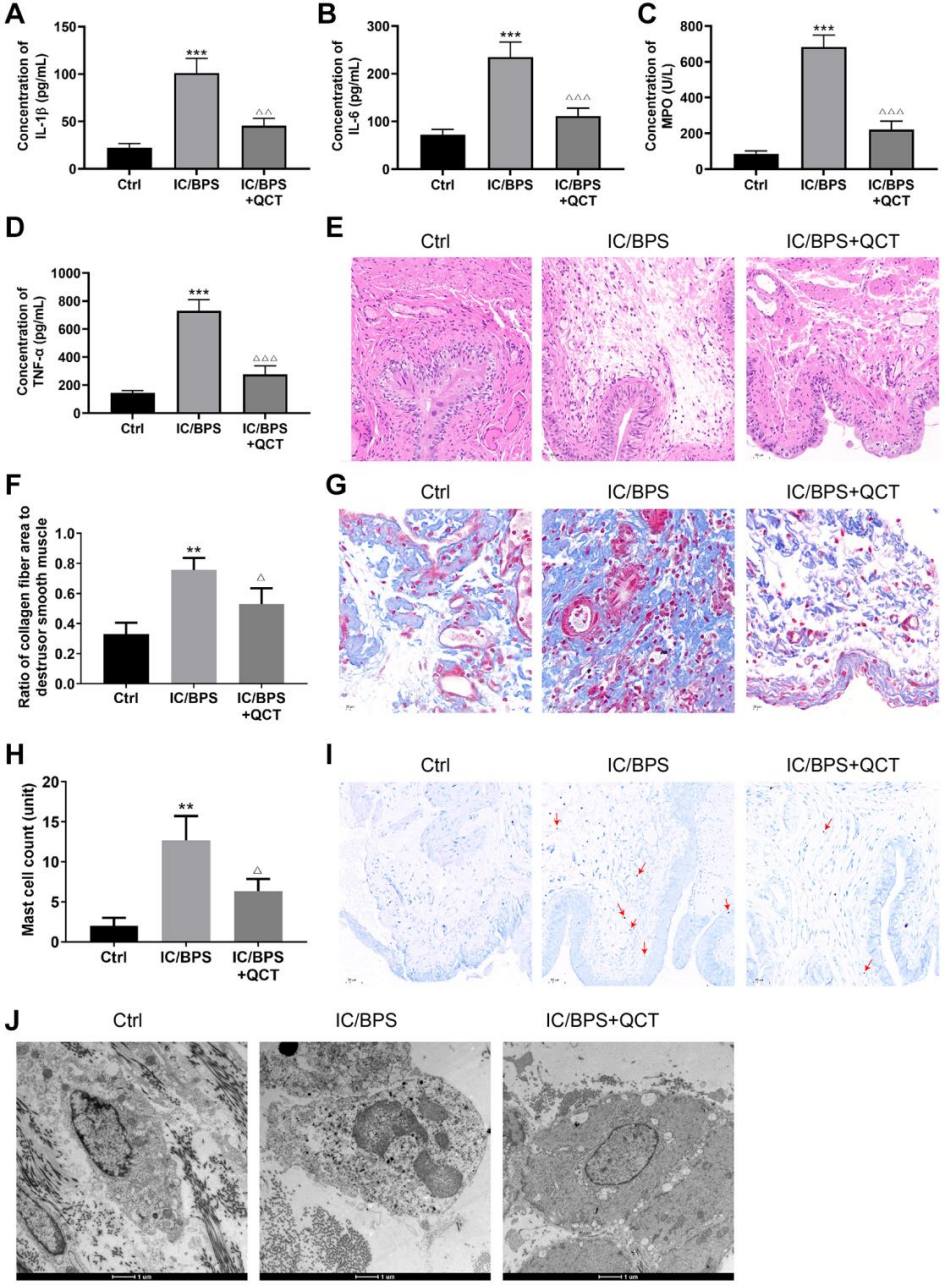
**The effect of QCT on IC/BPS rats.** ELISA kit detected the concentration of IL-1β (**A**), IL-6 (**B**), MPO (**C**), TNF-α (**D**). (**E**) HE staining was used to deserve the inflammatory of bladder tissue in rat. (**F**, **G**) Changes of collagen fibers in rat bladder tissue was performed by Masson staining. (**H**, **I**) TB staining observed the mast cells in bladder tissue of rat. (**J**) The degranulation of mast cells in rat bladder tissue was observed by transmission electron microscope. One-way ANOVA was used. Compared with Ctrl group, ^**^P < 0.01, ^***^P < 0.001. Compared with IC/BPS group, ^Δ^*P* < 0.05, ^ΔΔ^*P* < 0.01, ^ΔΔΔ^*P* < 0.001. *n* = 3.

Next, we observed the influence of QCT on IC/BPS model rats through experiments, and found that the level of IL-1β (Figure 2A), IL-6 (Figure 2B), MPO (Figure 2C) and TNF-α (Figure 2D) in the IC/BPS group increased compared with the control group. QCT treatment significantly inhibited the concentrations of IL-1β, IL-6, MPO and TNF-α in the serum of the IC/BPS rats. HE staining showed that LL-37 induced inflammatory infiltration of rat bladder tissue was significantly more than that of the control group (Figure 2E), and QCT reduced the inflammatory infiltration of IC/BPS rats. Masson and TB staining showed that the area of collagen fibers (Figure 2F, 2G) and the number of mast cells (Figure 2H, 2I) increased significantly in LL-37-induced rats, and the area of collagen fibers and the numbers of mast cells were inhibited by QCT treatment.

The results of the transmission electron microscope are shown in Figure 2J. In the bladder tissue of rats in the IC/BPS group, the mast cells infiltrated obviously, and the degranulated mast cells appeared vacancies. The particulate matter in the cytoplasm was released to the outside. The degranulation of mast cells was significantly improved in the QCT-treated group. The results demonstrated that QCT may alleviate LL-37-induced inflammation and the increase of collagen fibers and mast cells in rats.

### The expression of mRNA in different groups

**Figure 3:**
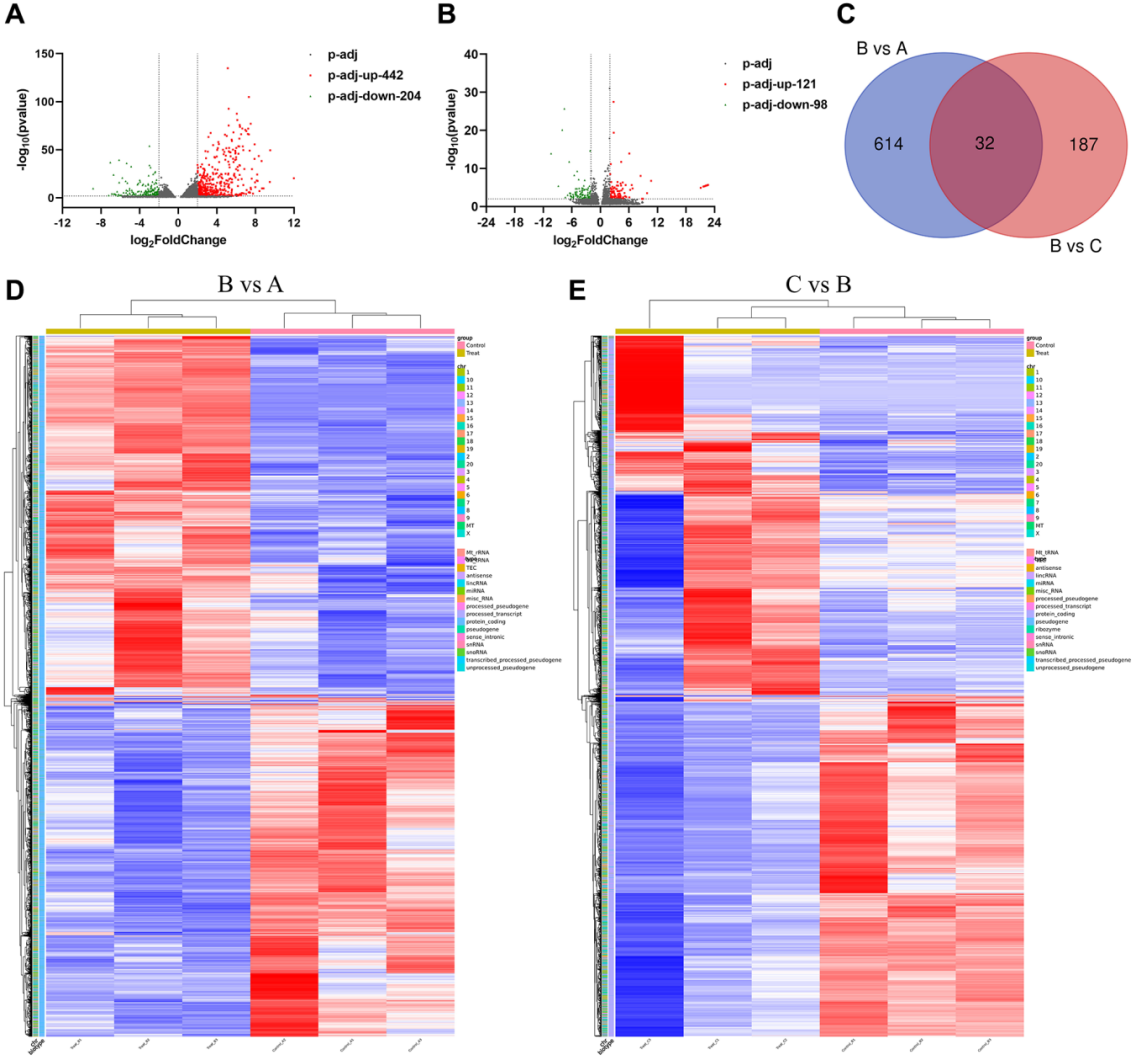
**The expression of mRNA in different groups.** (**A**) Volcano map of differential mRNA between B group and A group. (**B**) Volcano map of differential mRNA between B and C group. (**C**) Venn diagram of DE mRNAs in B vs. A and C vs. B group. (**D**) DE genes clustering heat map between B and A group. (**E**) Differential gene clustering heat map between B and C group. A: Ctrl group. B: IC/BPS group. C: IC/BPS+QCT group.

We collected the rat bladder tissue for mRNA sequencing, and found that there were 646 mRNAs differentially expressed (DE) (|log2 FC| ≥2, *P*.adj ≤0.01). Compared with the control group, there were 442 mRNAs up-regulated and 204 mRNAs down-regulated in the IC/BPS group (Figure 3A). Compared with IC/BPS group, there were 219 mRNAs DE in IC/BPS+QCT group, including 121 mRNAs up-regulated and 98 mRNAs down-regulated (Figure 3B). Tables 3 and 4 show the top 20 up-regulated mRNAs and top 20 down-regulated mRNAs. Venn analysis was performed on DE mRNA in the two comparison groups, there were 32 mRNAs co-expression in IC/BPS vs. control group and IC/BPS+QCT vs. IC/BPS group (Figure 3C). Clustering analysis was shown in heatmap (Figure 3D, 3E).

**Table 3:** Top 20 up-regulated and top 20 down-regulated mRNAs in IC/BPS group compared with Ctrl group.

**Gene**	**Log2 fold change**	***P*.adj**
**Up-regulation**		
Ly6al	11.9995	0.0000
Cenpf	9.5340	0.0000
Pbk	9.5013	0.0000
Xkr5	8.8418	0.0000
Kif14	8.6815	0.0000
Bub1b	8.6416	0.0000
Rrm2	8.4192	0.0000
Mmp7	8.3470	0.0000
Shcbp1	8.2388	0.0000
Melk	8.1987	0.0000
Troap	8.1382	0.0000
Ube2t	7.9999	0.0000
Cenpw	7.9709	0.0000
Cdca3	7.9262	0.0000
LOC103690108	7.9103	0.0000
Anxa10	7.7509	0.0000
Pimreg	7.6506	0.0000
Spc25	7.6189	0.0000
Pcsk9	7.5383	0.0000
Ccna2	7.5194	0.0000
**Down-regulation**		
Klkb1	−8.8226	0.0000
Oxtr	−7.1984	0.0026
Olig1	−7.0329	0.0000
Cnpy1	−6.8884	0.0000
AABR07010986.1	−6.7267	0.0001
Mageb16	−6.6985	0.0000
AABR07024468.1	−6.5756	0.0001
AABR07010985.1	−6.5422	0.0002
RGD1559441	−6.4515	0.0004
Tmem179	−6.3277	0.0000
Igh-6	−6.2578	0.0057
LOC108348172	−6.1880	0.0002
LOC100361008	−6.1273	0.0000
Pck1	−6.1071	0.0000
Bmp8a	−6.0903	0.0020
Cyp2f4	−5.9654	0.0016
Dhrs7c	−5.8553	0.0021
Slc18a3	−5.7973	0.0090
Tma7	−5.7955	0.0000
Plin1	−5.6610	0.0000

A: Ctrl group. B: IC/BPS group. C: IC/BPS+QCT group.

**Table 4:** Top 20 up-regulated and top 20 down-regulated mRNAs in IC/BPS+QCT group compared with IC/BPS group.

**Gene**	**Log2 fold change**	***P*.adj**
**Upregulation**		
Cd177	22.6521	0.0000
Astl	22.5311	0.0000
Smpx	22.3440	0.0000
Trim58	22.2868	0.0000
Myog	22.2132	0.0000
Hbe1	22.0227	0.0000
AABR07034455.1	21.9977	0.0000
Dand5	21.9072	0.0000
AABR07059632.2	21.8511	0.0000
Gcg	21.8511	0.0000
Tph2	21.8251	0.0000
Gal	21.7985	0.0000
Sst	21.7985	0.0000
Defb24	21.7664	0.0000
AC118963.2	21.7664	0.0000
Myod1	21.6541	0.0000
Saxo1	21.1000	0.0000
Lcn2	10.6896	0.0000
Sirpd	9.8326	0.0003
Orm1	8.9567	0.0093
**Down-regulation**		
Ly6al	−10.3506	0.0000
Lypd2	−8.8460	0.0000
LOC103690108	−8.0264	0.0000
Mmp7	−7.5516	0.0000
Sp8	−7.3866	0.0039
Noxo1	−6.8706	0.0010
Clca1	−6.5533	0.0000
Cldn3	−6.4390	0.0003
Gstm5	−6.2917	0.0012
Rnase1l1	−6.2557	0.0003
Tmem221	−6.1713	0.0019
Tex49	−6.1468	0.0072
Krt4	−5.9845	0.0025
AC128836.1	−5.7501	0.0007
Sult1c2a	−5.6673	0.0062
AABR07016779.1	−5.6662	0.0030
LOC100910438	−5.6292	0.0017
Crip3	−5.5480	0.0013
Tspan1	−5.4728	0.0000
Sphkap	−5.3190	0.0000

A: Ctrl group. B: IC/BPS group. C: IC/BPS+QCT group.

### Pathway enrichment analysis

**Figure 4:**
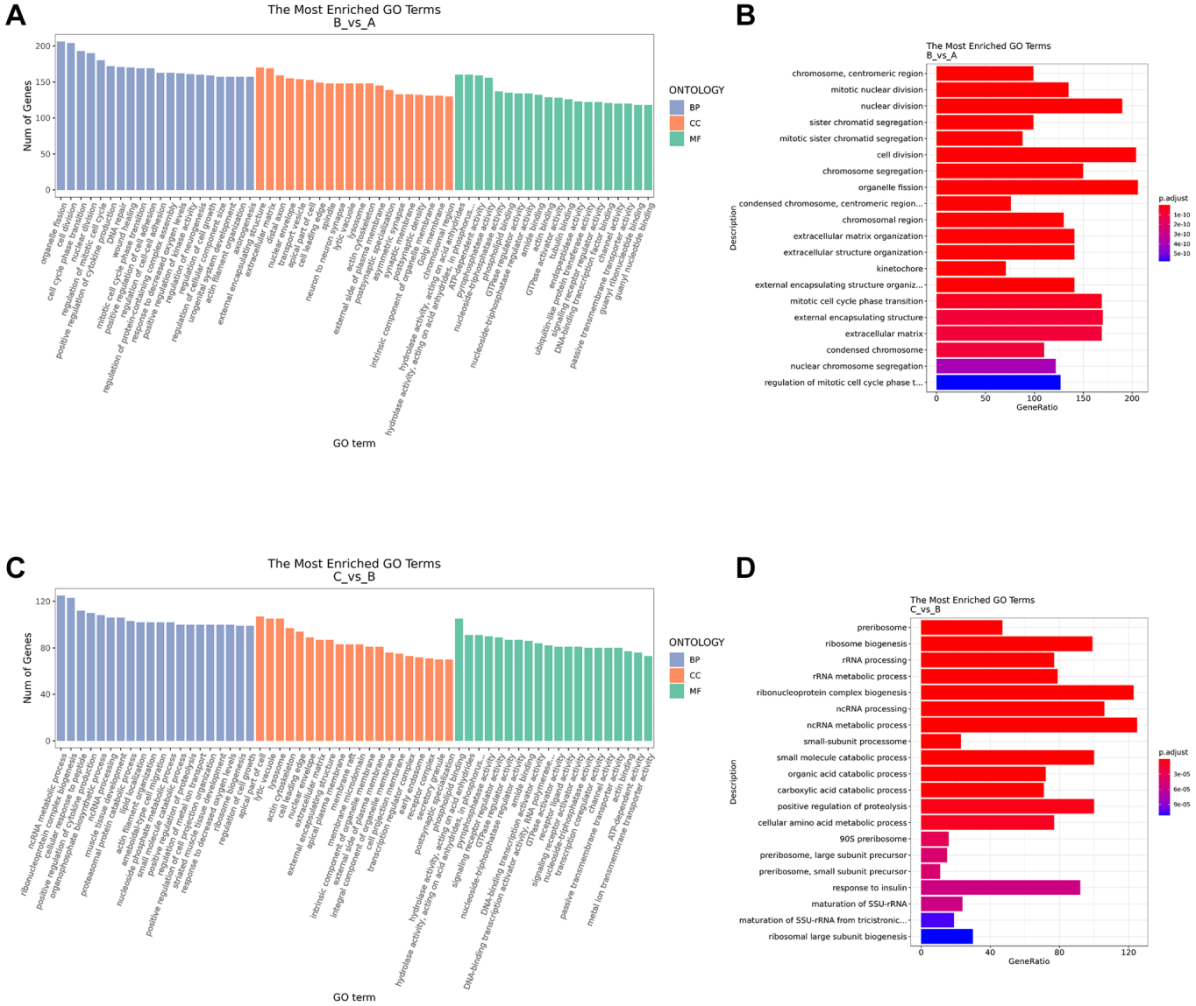
**GO function enrichment analysis.** (**A**) GO annotation analysis of differential mRNA in BP, CC, MF between B and A group. (**B**) Top 20 of GO annotation terms between B and A group. (**C**) GO annotation analysis of differential mRNA in BP, CC, MF between C and B group. (**D**) Top 20 of GO annotation terms between C and B group. A: Ctrl group. B: IC/BPS group. C: IC/BPS+QCT group.

In order to further study the biological function of DE mRNA, GO enrichment analysis was carried out. The top 20 enriched GO terms in BP, CC and MF were shown in Figure 4A. Compared with control group, the terms of DE mRNA in the IC/BPS group mainly included organelle fission, cell division, nuclear division, external encapsulating structure, mitotic cell cycle phase transition, extracellular matrix, chromosome segregation, extracellular matrix organization, extracellular structure organization, external encapsulating structure organization (Figure 4B). Compared with the IC/BPS group, the GO terms of DE mRNA in the IC/BPS+QCT group mainly included ncRNA metabolic process, ribonucleoprotein complex biogenesis, ncRNA processing, small molecule catabolic process, positive regulation of proteolysis, ribosome biogenesis, response to insulin, rRNA metabolic process, rRNA process, cellular amino acid metabolic process (Figure 4C, 4D).

**Figure 5:**
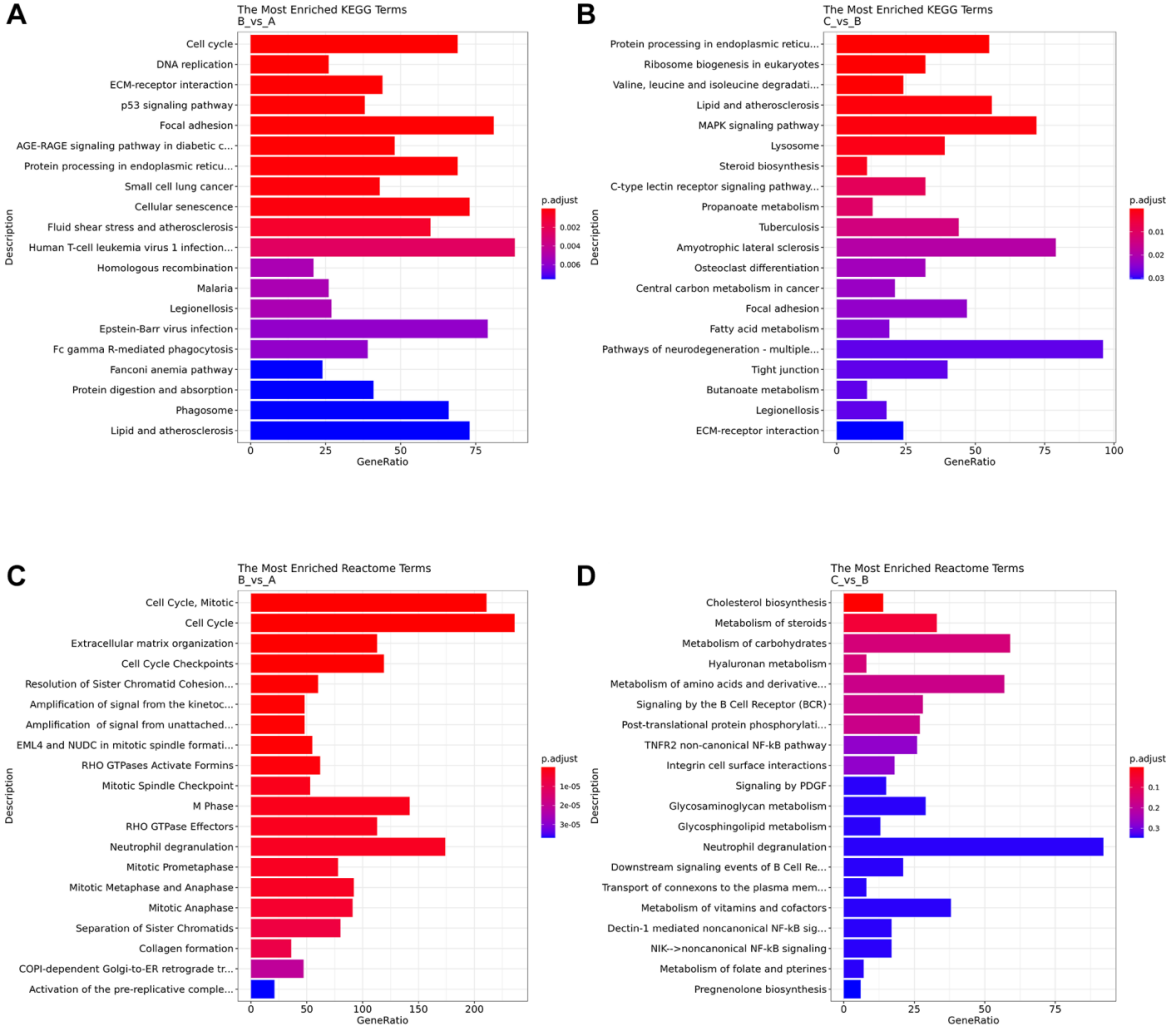
**Analysis of pathway enrichment.** (**A**) KEGG analyzed the top 20 enriched pathway between B and A group. (**B**) KEGG analyzed the top 20 enriched pathway between B and C group. (**C**) Reactome analyzed the top 20 enriched pathway between B and A group. (**D**) Reactome analyzed the top 20 enriched pathway between B and C group.

KEGG analysis showed that compared with the control group, human T-cell leukemia virus 1 infection pathways, focal adhesion, Epstein-Barr virus infection, cell cycle, cellular senescence were enriched in the DE mRNA of the IC/BPS group (Figure 5A). Compared with IC/BPS group, pathways of neurodegeneration - multiple diseases, amyotrophic lateral sclerosis, MAPK signaling pathway, protein processing in endoplasmic reticulum, lipid and atherosclerosis pathway were significantly enriched in the IC/BPS+QCT group (Figure 5B).

Reactome pathway analysis showed that cell cycle, “cell cycle, mitotic”, neutrophil degranulation, M Phase, cell cycle checkpoints, extracellular matrix organization were enriched in DE mRNAs of the IC/BPS (Figure 5C). And neutrophil degranulation, metabolism of carbohydrates, “metabolism of amino acids and derivatives”, “metabolism of vitamins and cofactors” were significantly enriched in DE mRNAs of the IC/BPS+QCT group (Figure 5D).

### PPI network construction and the most significantly modules of genes

**Figure 6:**
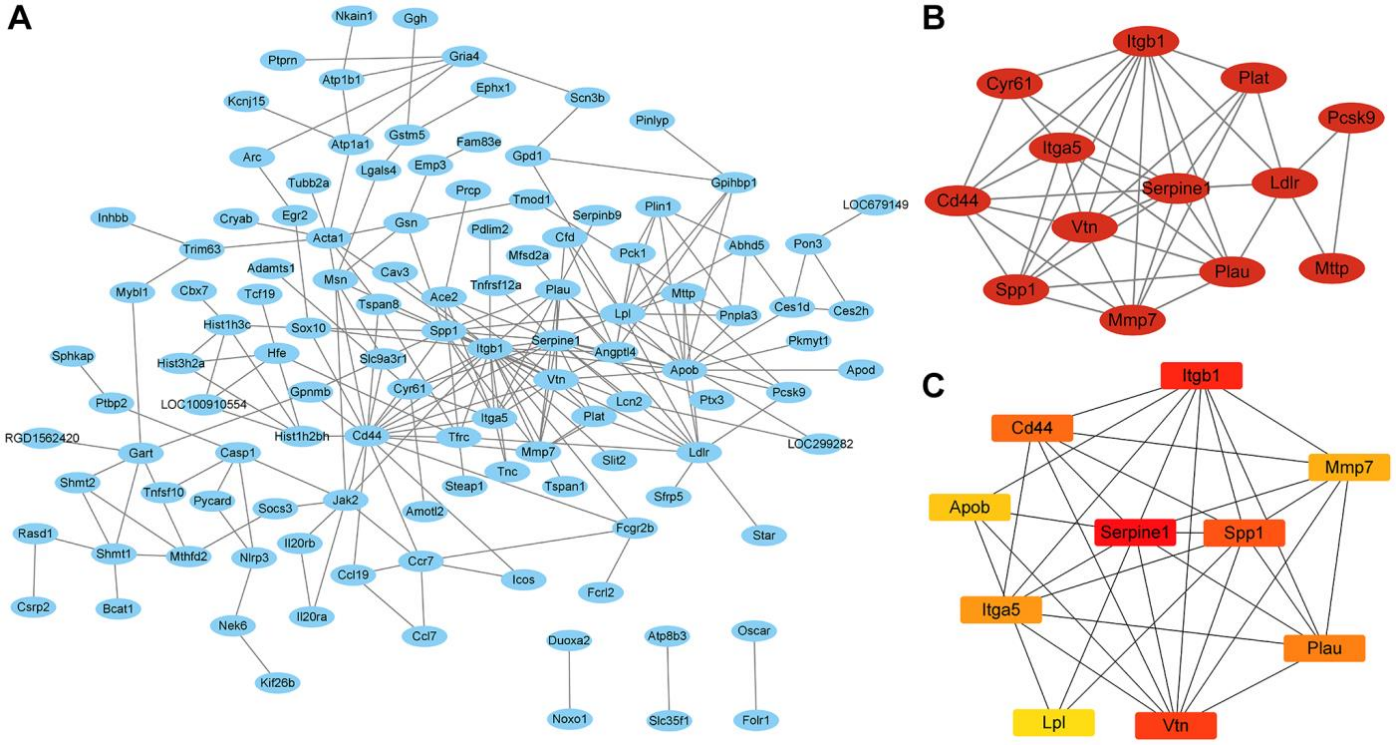
**The PPI network and the most significant modules of genes.** (**A**) PPI network was analyzed in B vs. A group and B vs. C group (|log2 Fold change| ≥ 1 and *P*.adj <0.05) by STRING software. (**B**) The most significant module was identified by Cytoscape MCODE (degree cutoff = 2, node score cutoff = −0.2, k-core = 2, and max. Depth = 100). (**C**) The top 10 hub genes were identified by Cytoscape Cytohubba.

We intersected the mRNA that was DE at the same time in the IC/BPS vs. control group and the IC/BPS+QCT vs. IC/BPS group (|logFC| > 1 and *P*.adj <0.05). A total of 179 mRNAs were imported into the PPI network using STRING, there were 179 nodes and 435 edges in the PPI network (Figure 6A). We applied Cytoscape MCODE for further analysis and found that 13 central nods (Cyr61, Cd44, Spp1, Mmp7, Plau, Ldlr, Pcsk9, Mttp, Plat, Itgb1, Itga5, Vtn, Serpine1) formed the significantly modules among 179 nodes (Figure 6B). The hub gene of was confirmed by Cytobubba (a plug-in Cytoscape). The top ten genes included Serpine1, Itgb1, Vtn, Spp1, Cd44, Plau, Itga5, Mmp7, Apob and Lpl (Figure 6C). Among them, Serpine1, Spp1, Itga5, Mmp7 and Lpl were co-expressed in IC/BPS+QCT group and IC/BPS group (Table 5).

**Table 5:** Hub genes identified by Cytohubba.

**Gene**	**Score**	**IC/BPS vs. Ctrl group**		**IC/BPS+QCT vs. IC/BPS group**	
		**log2 fold change**	***P*.adj**	**log2 fold change**	***P*.adj**
Serpine1	628	2.0883	0.0000	4.3945	0.0000
Spp1	497	4.1380	0.0000	3.8057	0.0113
Itga5	272	1.6057	0.0007	1.8988	0.0432
Mmp7	270	8.3470	0.0000	−7.5516	0.0000
Lpl	82	−1.4981	0.0059	2.4786	0.0101

### Lpl involved in the therapeutic process of QCT on IC/BPS rats

**Figure 7:**
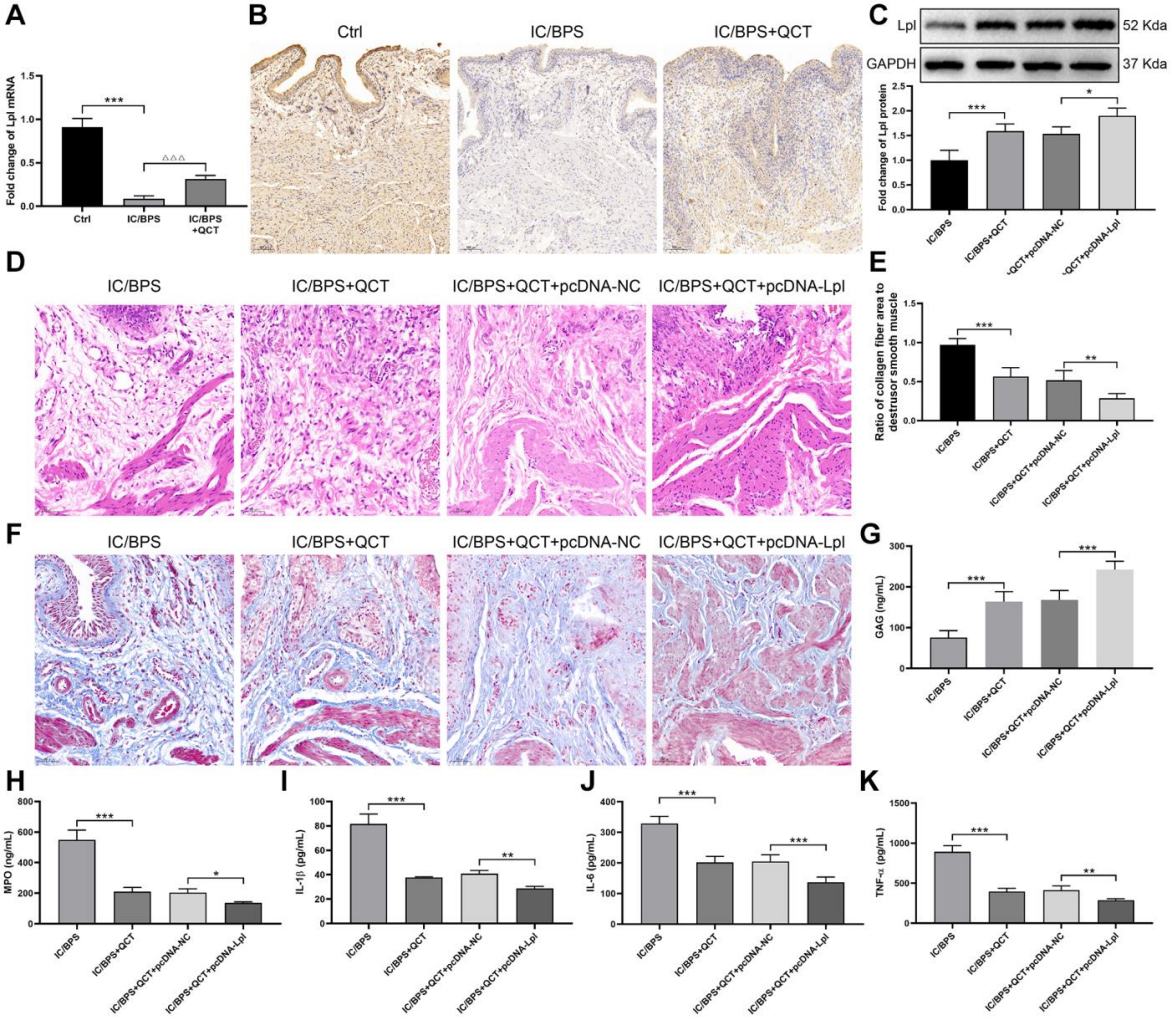
**Lpl was involved in the therapeutic process of QCT on IC/BPS in rats.** (**A**) Lpl mRNA expression was detected using RT-qPCR. (**B**) The IHC staining of bladder tissues. (**C**) The protein expression of Lpl. (**D**) The HE staining of bladder tissues. (**E**, **F**) Masson staining was used to evaluate the collagen and smooth muscle in bladder tissues. The levels of GAG (**G**), MPO (**H**), IL-1β (**I**), IL-6 (**J**), TNF-α (**K**). ^*^P < 0.05, ^**^P < 0.01, ^***^P < 0.001.

According to Cytobubba’s prediction and mRNA sequencing results, we chose Lpl for further study. RT-qPCR results indicated that Lpl mRNA was decreased in IC/BPS group (Figure 7A), QCT treatment promoted Lpl expression. IHC staining displayed the same results with RT-qPCR (Figure 7B). Next, we further explore the regulatory effect of Lpl on IC/BPS rats. We injected Lpl overexpression plasmid and its negative control into rats through tail vein, and found that Lpl protein was significantly upregulated in Lpl overexpression group (Figure 7C). Pathological staining showed that compared with QCT+pcDNA-NC group, the infiltration of inflammatory cells in the overexpressed Lpl group was less than that in the control group, the cells were arranged neatly (Figure 7D) and the collagen fibers in the tissue were reduced (Figure 7E, 7F). ELISA kits were used to examine the level of GAG, MPO, IL-1β, IL-6 and TNF-α. And the results revealed that overexpression of Lpl increased GAG level in the bladder tissue of rats (Figure 7G). However, MPO, IL-1β, IL-6 and TNF-α were depleted in Lpl overexpression group (Figure 7H–7K). These results demonstrated that treatment with QCT upregulated Lpl expression, increased Lpl relieved the development process of IC/BPS.

## DISCUSSION

It is reported that QCT has a variety of biological activities, such as anti-inflammation [13], anti-cancer [14], anti-oxidation [15], and inhibition osteoclast differentiation [16], and it has regulatory effects on many diseases. For example, QCT promoted the apoptosis and pyroptosis of lung cancer cells through the SIRT1/AMPK signal pathway [17]. QCT could stimulate the generation of osteoblast markers and calcified nodules and promote the osteogenic differentiation of bone marrow mesenchymal stem cells [16]. QCT reduced the free radical oxidation in cardiac mitochondria of type 2 diabetes mellitus rats, improved the oxidative stress of cardiomyocytes in rats and reduced cardiovascular risk in patients with type 2 diabetes mellitus [18]. In the current study, we found that QCT significantly reduced the increase of pro-inflammatory cytokines in rat plasma and damage to bladder tissue induced by LL-37.

In order to study the effects of QCT on LL-37-induced IC/BPS model rats, we first determined the concentration of QCT in plasma and urine of QCT-treated IC/BPS rats by HPLC, and confirmed that QCT relieved the symptoms of IC/BPS rats by ELISA and HE/MASSON and TB staining tests. Subsequently, we performed the mRNA sequence of bladder tissue in rats and identified 219 DE mRNA with significant expression after treatment with QCT. Subsequently, through enrichment analysis including GO terms enrichment analysis, KEGG pathway analysis, Reactome pathway analysis, PPI network analysis, we found the DE mRNAs and signaling pathways associated with the treatment of IC/BPS by QCT could affect the development of the disease in many ways.

GO analysis showed that DE mRNAs were mainly enriched in ncRNA metabolic process, ribonucleoprotein complex biogenesis, ncRNA processing, small molecule catabolic process, positive regulation of proteolysis. KEGG pathway analysis revealed that pathways of neurodegeneration - multiple diseases, amyotrophic lateral sclerosis, MAPK signaling pathway were significantly enriched. The Reactome pathway analysis results were enriched in neutrophil degranulation, metabolism of carbohydrates, metabolism of amino acids and derivatives.

STRING software was used to construct PPI interaction network. We identified the Hub gene through Cytoscape plug-in Cytohubba, and finally screened out five genes (Serpine1, Spp1, Itga5, Mmp7 and Lpl) that were both expressed in the IC/BPS and IC/BPS+QCT groups. Serpin family E member 1 (Serpine1) is the principal inhibitor of tissue plasminogen activator and urokinase and encodes a member of a superfamily of serine protease inhibitors. Serpine1 has been found to be closely related to thrombosis [19], tumor progression [20], immune escape [21], and inflammatory response [22]. Spp1 (Secreted Phosphoprotein 1) is a protein-encoded gene that encodes the protein involved in osteoclast differentiation and attachment of mineralized bone matrix. In addition, SPP1 is involved in the regulation of cell proliferation [23], metastasis [24], drug resistance [25], immune infiltration [26], osteogenic differentiation [27]. Itga5 (Integrin Subunit Alpha 5) is a receptor for fibronectin and fibrinogen, and the product of the gene is the integrin alpha chain family. At present, ITGA5 has been reported to have poor prognosis in many tumors including glioma [28], pancreatic cancer [29], Oral Squamous Carcinoma [30], bladder cancer [31]. Lee et al. [32] reported that the expression level of ITGA5 was increased in patients with atopic dermatitis, and inhibition of ITGA5 could alleviate skin inflammation. Matrix Metallopeptidase 7 (Mmp7), a member of the MMP family, is the main protease for extracellular matrix degradation. MMP7, a member of the MMP family, is the main protease for the degradation of ECM. The differential expression of MMP7 plays an important role in many diseases. For example, MMP7 promoted epithelial-mesenchymal transition of prostate cancer by promoting IL-17 expression [33]. MMP7 was highly expressed in colon cancer tissues and was associated with poor prognosis of patients [34]. MMP7 was independently associated with psoriasis and inflammatory responses, and could be used as a potential biomarker for psoriasis [35].

Lipoprotein lipase (Lpl) is a glycoprotein secretase that catalyzes the hydrolysis of triglycerides in circulating chylomicrons and very low-density lipoproteins [36]. Studies have shown that inactivation or mutations of Lpl could lead to the occurrence of a variety of diseases [37, 38], such as hypertriglyceridemia and acute pancreatitis [39], prostate cancer [40], Alzheimer’s disease [41] and so on. Lpl acted as a bridging molecule not only cell-surface and lipoproteins but also cell-surface GAGs and Aβ and the cellular uptake of Aβ in astrocyte [42]. Here, we revealed that Lpl was decreased in IC/BPS rats, QCT treatment promoted the Lpl expression. Overexpression of Lpl promoted GAG production, and repressed the level of MPO, IL-1β, IL-6, TNF-α concentrations, and then alleviated the IC/BPS. In addition, loving et al. reported that Lpl inhibition promoted the polarization of microglia into pro-inflammatory state and resulted in lipid droplets accumulation [43].

In summary, in the study, we revealed that QCT significantly improved the LL-33-induced inflammatory response and bladder injury in the IC/BPS rat model. mRNA sequencing showed that there were significant differences in the expression of mRNAs in bladder tissues between QCT treated group and the untreated group. Cytohubba screened out the hub genes Serpine1, Spp1, Itga5, Mmp7 and Lpl. QCT upregulated the expression of Lpl. Overexpression of Lpl promoted GAG level and relieved bladder injury in rats through inhibiting the production of pro-inflammatory cytokines. However, more regulatory mechanisms of QCT-mediated IC/BPS still need to be studied in future.
